# Combined effects of flow condition and parasitism on shoaling behaviour of female guppies *Poecilia reticulata*

**DOI:** 10.1007/s00265-014-1760-5

**Published:** 2014-06-29

**Authors:** F. A. Hockley, C. A. M. E. Wilson, N. Graham, J. Cable

**Affiliations:** 1 School of Biosciences, Cardiff University, Cardiff, UK; 2 Hydro-environmental Research Centre, School of Engineering, Cardiff University, Cardiff, UK

**Keywords:** Cohesion, Flow rate, Flume, Nearest neighbour distance, Shoal size, Social groups

## Abstract

Group living in fish can provide benefits of protection from predators and some parasites, more efficient foraging for food, increased mating opportunities and enhanced energetic benefit when swimming. For riverine species, shoaling behaviour can be influenced by various environmental stressors, yet little is known how flow rate might influence the shoaling of diseased fish shoals. In view of the increasingly unpredictable flow rates in streams and rivers, this study aimed to assess the combined effect of flow condition and parasitism on the shoaling behaviour of a model fish species. Shoal size, shoal cohesion and time spent shoaling of female guppies *Poecilia reticulata* were compared when infected with the directly transmitted ectoparasite *Gyrodactylus turnbulli* under flow and static conditions. Flow condition was an important factor in influencing shoaling behaviour of guppies with the fish forming larger shoals in the absence of flow. When a shoal member was infected with *G. turnbulli*, shoal cohesion was reduced, but the magnitude of this effect was dependent on flow condition. In both flow and static conditions, bigger fish formed larger shoals than smaller counterparts. Future changes to stream hydrology with more frequent flooding and drought events will affect the shoaling tendency of fish. During high-flow events, diseased fish may not be able to keep up with shoal mates and therefore have a higher risk of predation. Additionally, these findings may be important for aquaria and farmed species where an increase in flow rate may reduce aggregation in fish.

## Introduction

Many fish spend part or all of their lifetime living in groups forming either loosely aggregated shoals or polarised synchronised schools (Pitcher and Parrish 1993; Stumbo et al. 2012). The costs and benefits of grouping behaviour, environmental influences on shoaling and social interactions within shoals have been widely studied by animal ecologists (Pitcher and Parrish 1993). The physical constraints of being able to observe the natural behaviour of fish or recapture individuals in the wild have led to many laboratory or mesocosm experiments. Such experiments, by design, remove complexity from the study system to concentrate on a small number of potentially influencing factors. This has often resulted in shoaling studies using standard fish tanks with minimal water movement which do not reflect the flow conditions that fish experience in the wild.

Considering that riverine fish are subjected to a variety of flow conditions, from large-scale tidal or riverine currents to small-scale microhabitat variations, surprisingly few studies have investigated how shoaling preferences differ under different magnitudes of flow. In a recent study, Chicoli et al. (2014) demonstrated that shoal structure, stimulus detection and transmission of information between shoal members differed between flow and no-flow conditions in a small laboratory flume. Garner (1997) found that minnows (*Phoxinus phoxinus*) shoaled less during a high flow rate and the shoals failed to return to their original size within an hour following the flow event. Dominance hierarchies of three-spined stickleback (*Gasterosteus aculeatus*) shoals became increasing unstable as flow rate increased (simulating spate conditions) or water levels decreased (simulating drought) (Sneddon et al. 2006). In contrast, juvenile chub (*Leuciscus cephalus*) shoaling was not affected by flow rate unless in the presence of a predator where they exhibited greater aggregation at a higher flow rate (Allouche and Gaudin 2001). This indicates that shoaling differences with respect to flow could also depend on other influencing abiotic or biotic factors.

Abiotic influences known to affect shoaling include temperature (Weetman et al. 1999), vegetation cover (Allouche and Gaudin 2001) and demography (Song et al. 2011), but there are also many biotic factors which influence shoaling including sex (Griffiths and Magurran 1998; Ruhl and McRobert 2005; Richards et al. 2010), size (Paxton 1996; Hoare et al. 2000a) and disease (reviewed by Barber et al. 2000). Fish shoals tend to be size-assorted to minimise phenotypic oddity (Hoare et al. 2000a), and there is evidence for an increase in shoaling tendency with larger fish (Pitcher et al. 1983; Paxton 1996; Hoare et al. 2000a) due to their higher conspicuousness, higher calorific value to predators (Rodgers et al. 2011), better foraging ability (Hoare et al. 2000a) and development of discriminatory ability by larger fish to select larger shoals (Ledesma and McRobert 2008).

Previous studies have shown that parasites such as *Gyrodactylus turnbulli* on guppies (*Poecilia reticulata*) can influence population dynamics, as demonstrated in both laboratory and mesocosm experiments (Richards et al. 2010, 2012; Croft et al. 2011). When considering infectious disease, the costs and benefits of group living can be complex, typically varying with host taxa and the mode of parasite transmission. For parasites which actively seek out their hosts, the benefits of shoaling can be similar to that of antipredator defence. The larger the group size, the lower the chance of a particular individual becoming infected (Poulin and Fitzgerald 1989). However, for directly transmitted parasites, host-host contact within a social group could increase the spread of disease and therefore cause avoidance behaviour of group members. For example, three-spined sticklebacks avoided shoaling with conspecifics infected with the ectoparasite *Argulus canadensis* in a choice experiment (Dugatkin et al. 1994). Avoidance behaviour has also been observed for hosts infected with parasites with indirect lifecycles; for example banded killifish (*Fundulus diaphanous*) chose to shoal with unparasitised groups over those that were infected with the trematode *Crassiphiala bulboglossa* (see Krause and Godin 1996). However, it is likely that often the antipredator and foraging benefits of group living will outweigh the risk of infection (Barber et al. 2000). Some studies have even shown infected fish to shoal more. For example, rainbow trout (*Oncorhynchus mykiss*) infected with the trematode *Diplostomum spathaceum* showed enhanced shoaling tendencies compared to uninfected controls (Mikheev 2009).

Of the few studies which have examined the effect of flow rate on host-parasite interactions, generally, there is a higher prevalence and intensity of parasites in low-flow conditions. This may be due to an increased rate of contact between parasite and host and reduced physiological condition of the host due to increased sedimentation, hypoxia and lower food availability making them more susceptible to infectious disease (Leniham et al. 1999) or due to infected fish having lower energy reserves so selecting areas with low flow. For example, American eels *Anguilla rostrata* had higher prevalence and intensity of *Ergasilus celestis* and *Pseudodactylogyrus anguillae* at a water velocity <5 cm s^−1^ (Barker and Cone 2000), and oysters *Crassostrea virginica* experienced a higher prevalence and intensity of the protist *Perkinsus marinus* at the base of reefs where flow rates were lower (Leniham et al. 1999). Similarly, free-living infective stages of *Myxobolus cerebralis* were at a higher density in a low-flow system (water velocity 0.02 cm s^−1^) compared to a relatively faster flow system (water velocity 2 cm s^−1^) and this was also reflected in the infection prevalence and duration in rainbow trout fry *O. mykiss* (see Hallett and Bartholomew 2008). High flow rates lowered mortalities in channel catfish (*Ictalurus punctatus*) associated with *Ichthyophthiruis multifiliis* (see Bodensteiner et al. 2000). In mammalian hosts, the highest infectivity of *Schistosoma mansoni* cercariae was detected at water velocities of 30–40 cm s^−1^. Infections were reduced at velocities >40 cm s^−1^ due to increased turbulence affecting cercarial penetration and at <30 cm s^−1^ perhaps due to fewer contacts between the parasite and hosts (Sousa and Grosholz 1991). At present, such data is difficult to interpret as little is known about the changes in infected host behaviour with respect to flow (Hockley et al. 2014). The changes in host behaviour would in turn affect transmission potential of directly transmitted parasites and therefore better explain the reasons for the increase in parasitism at low flows. The combined effect of parasitism and flow on shoaling behaviour of fish, to our knowledge, has never been investigated.

In this study, we assess how the addition of flow affects the tendency of fish to shoal compared to the static conditions typically used in laboratory experiments. We also investigate how the size of the fish and parasitism influences shoaling in each condition. We use a common host-parasite model system guppies *P. reticulata* and the directly transmitted ectoparasitic monogenean *G. turnbulli*. Guppies are a popular model species in ecological and evolutionary studies, and their shoaling behaviour is particularly well studied (Magurran 2005). Previous studies have shown that parasites such as *G. turnbulli* can influence population dynamics, as demonstrated in both laboratory and mesocosm experiments (Richards et al. 2010, 2012; Croft et al. 2011). It is predicted that shoaling will be reduced by both an increased flow rate and the presence of an infected individual and that larger fish will have a higher tendency to shoal.

## Methods

### Study system

Experiments were conducted between January and April 2012 using offspring from wild-caught guppies *P. reticulata*. Guppies were originally caught in the Tunapuna River, Trinidad, and maintained in aquarium facilities at Bristol University, UK, before being transferring to the School of Biosciences, Cardiff University, UK, in October 2005. Female guppies (13.6–27.3-mm standard length) were size matched in groups of six individuals. Each shoal was housed in a 6-L aerated tank for a minimum of 12 days to familiarise (Griffiths and Magurran 1997) with different shoals visually isolated from each other. The shoals were maintained under a 12-h light/12-h dark regime at a varying temperature range of 22–24 °C, fed on a diet of fish flakes (Aquarian®) and bloodworm, with half water changes every second day. An isogenic strain of *G. turnbulli* (Gt3) isolated from ornamental guppies in 1997 was used for all experimental infections.

### Experimental design

Observations of shoaling behaviour took place in a glass-walled unidirectional recirculating open-channel flume in the Hydro-environmental Research Centre (HRC), Cardiff University, UK. The channel measured 10-m length by 0.29-m width and had a tailgate weir at the downstream end to allow for control of the surface water profile. Discharge was adjusted by controlling the power provided to the pump through a control box, and 0.2-m thick honeycomb flow straighteners were used at both ends of the flume. The channel was set at a negative gradient of 1 in 1,000. Uniform flow was established by measuring the flow depths at nine points along the channel and calculating total energy of flow (*E*) using the equation *E = z + y + u* ^*2*^ */*2 *g* where *y* is the flow depth (0.135 m), *u* is the area mean velocity (*u = Q/A*), *g* is acceleration due to gravity, *Q* is discharge and *A* is the cross-sectional flow area of the channel. Energy lines were then generated for each weir setting and uniform flow defined as where the slope of the total energy line equalled the channel bed slope. Chlorides were removed from the water by the addition of Haloex at 0.02 ml L^−1^, and water was heated between the range of 24–26 °C using a 3-kW Electro Titanium Digital heater. A 2-cm^2^ grid was drawn on one side of the flume window in order for the observer to estimate nearest neighbour distance between the shoaling fish.

A static (no flow) and flow (flow action) condition with an area mean velocity of 0.125 ms^−1^ (discharge 0.0048 m^3^s^−1^) was chosen for the trials. The flow action chosen was similar in velocity to that found in guppy streams in Trinidad (Reznick et al. 2001). Both conditions had a uniform flow depth of 0.135 m along the channel. Ten shoals of six female fish were used in the flow action trial, and 13 shoals used in the static condition trial. On day 1, each shoal was acclimatised in the flume for 30 min where they were observed to exhibit normal behavioural before pre-infection trials took place. A focal fish from the shoal of six was randomly assigned, and then observed for a total of 10 min. During the first 5 min, the nearest neighbour distance (NND) ± 1 cm and size of shoal (number of fish in shoal) was recorded every 10 s for the focal fish. During the second 5-min period, the total time the focal fish spent shoaling was recorded. These observations were then repeated for two randomly selected non-focal fish from within the shoals. Shoaling was defined as being within four body lengths of the nearest neighbour (Pitcher et al. 1983), and NND was measured to a maximum of 20 cm, with distances any greater not scored. The identity of the focal fish was retained by a second observer carefully following that fish for the duration of the trial.

On completion of the first flume trials, six focal fish from the flow action trials and eight focal fish from the static condition were infected with *G. turnbulli* following standard procedures (e.g. Richards et al. 2010). This was achieved by anaesthetising the individual with 0.02 % MS222 and bringing it into contact with a heavily infected donor fish until four worms had transferred from donor to recipient (taking <5 min). Transfer of the parasites was observed continuously under a stereo-microscope. The other six focal fish from the flow action trials and five focal fish from the static condition trials acted as controls and were sham infected by placing them under anaesthetic and manipulating them under a microscope without transfer of parasites. Non-focal fish in all shoals were also sham-infected so all fish experienced the same degree of handling. All fish were then housed individually in 1-L pots to allow the infection to develop for 3 days but to prevent parasite transfer between fish. Although physically isolated, shoal members remained in visual contact throughout to maintain familiarity. The shoal groups were isolated from other shoals throughout the duration of the study.

On day 4, infection was confirmed by restraining each individual in a small amount of water in a crystalizing dish under a stereo-microscope. All uninfected fish were sham-screened to maintain equal handling stress. The shoals were then placed back in the open-channel flume for a repeated behavioural trial post-infection. The 10-min observations for the focal and two randomly chosen non-focal fish were repeated after 30 min of acclimatisation period. On completion of the trials, all fish were screened under anaesthetic for any parasite transmission and accurate worm counts. Mean intensity of *G. turnbulli* was 11.4 (range 1–29) after a 3-day infection. These comparatively low numbers are reflective of burdens observed in the wild (Harris and Lyles 1992; Fraser et al. 2010). There was no evidence of secondary pathology (e.g., fin clamping) in the fish at any time during the experiment, and there was no evidence of parasite transmission between fish during the 30-min post-infection trials.

### Statistical analysis

The effect of flow condition, parasitism and host standard length on shoaling behaviour parameters (shoal cohesion, shoal size and proportion of time shoaling) was assessed using generalised linear mixed models (GLMMs) with model selection and model averaging based on corrected Akaike Information Criterion (AICc) using the lme4 library (Bates et al. 2013) within the R statistical interface (R Core Team 2013) based on methods described in Burnham and Anderson (2002).

The model-fixed effects were shoal infection status (binary factor whether there was a member of the group infected or uninfected), individual infection status (binary factor whether the individual fish was infected or uninfected), flow condition (flow action or static) and fish standard length (mm). Two-way interactions were also included to account for any differences in parasitism and standard length in different flow conditions. Individual infection status was nested within group infection status as a random term. As each fish was tested twice, the identity of individual fish was included in the models as a random factor to account for repeated measures. Time of day (to the nearest hour) was also included as a random term to account for any temporal effects of shoaling.

Shoal cohesion (measured as the mean nearest neighbour distance, NND up to 20 cm) and mean shoal size (number of fish in shoal) were log_e_ + 1-transformed to normalise the data. The shoal cohesion GLMM was fitted using an inverse-Gaussian error structure and log link function, the shoal size GLMM was fitted using a Gaussian error structure with square root link function and the proportion of time shoaling fitted with a binomial error structure and logit link function. The fixed and random effects were included in global GLMMs with all plausible explanatory variables and interactions. Because the fixed effects were measured on different scales, the variables were standardised using the arm library (Gelman and Su 2013). The most important explanatory variables were determined by selecting the top most plausible models which fell within 2.5 AICc of the best model and model averaging the top models using the MuMIn library (Barton 2013) as described by Grueber et al. (2011). The output then gives the relative importance of each explanatory variable which is the sum of the Akaike weights for each variable for the models in which it appears across the top models, with the higher value (closest to 1) giving a higher relative importance compared to the other variables (Burnham and Anderson 2002). The variables were considered significant if the 95 % confidence intervals did not bound zero.

## Results

**Fig. 1:**
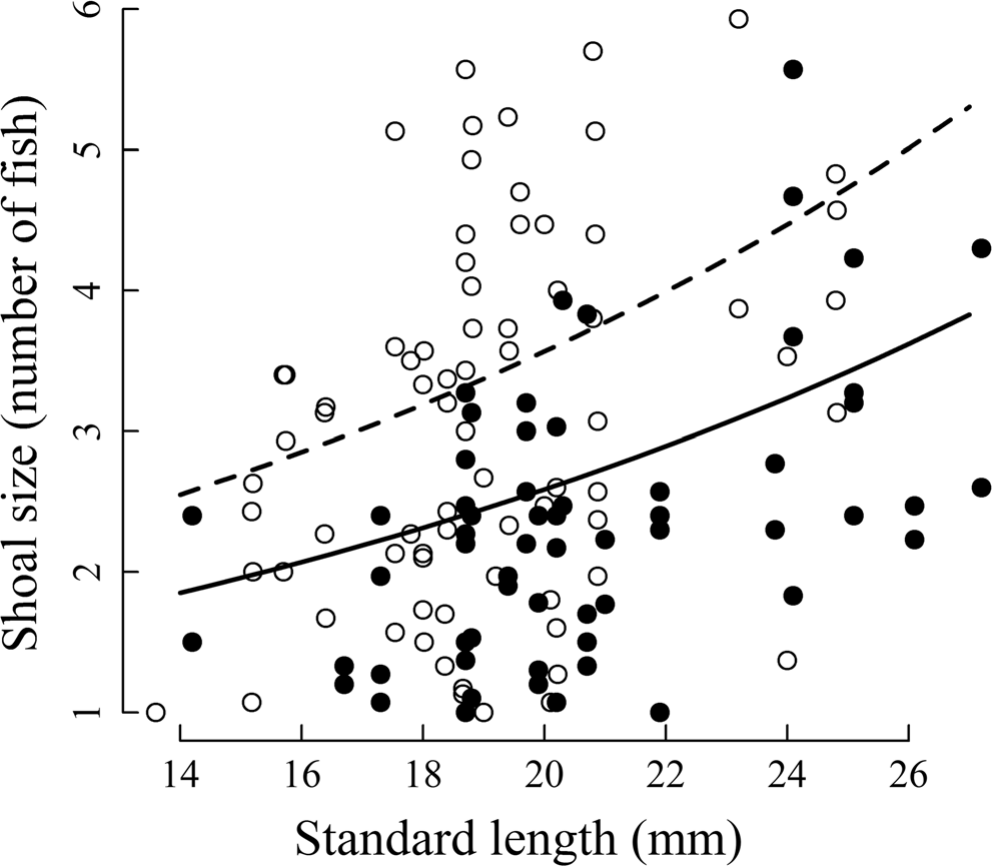
Size of guppy *Poecilia reticulata* shoals in flow (*closed circles*) and static (*open circles*) conditions. The predicted data trends generated by the top-scoring generalised linear mixed model are shown for flow (*solid line*) and static (*dashed line*) conditions

Flow condition was an important predictor for the size of *P. reticulata* shoals, with significantly larger shoals being formed in static conditions (mean 2.99, SE 0.15 fish) compared to the flow action condition (mean 2.35 SE 0.13 fish) (Table 1, Fig. 1). Additionally, the size of the fish was an important predictor of shoal size, with a significant positive correlation between fish standard length and number of individuals in the group (Table 1, Fig. 1).

**Table 1:** Summary of the averaged model predictors standardised to a mean of 0 and standard deviation 0.5 in shoaling behaviour of female guppies *Poecilia reticulata*. Averaged model is based on the top models with ∆AICc < 2.5 (averaged models based on four top models for shoal cohesion, five top models for shoal size and nine top models for proportion of time shoaling)

Dependent Variable	Predictor^ab^	Standardised estimate	Unconditional standard error	95 % confidence intervals ^c^	Relative importance
Shoal cohesion (nearest neighbour distance)	(Intercept)	0.225	0.126	(−0.023–0.473)	
	Flow	−0.017	0.074	(−0.163–0.128)	0.90
	Individual infected	−0.011	0.105	(−0.196–0.217)	0.90
	Flow: individual infected	−0.302	0.149	(−0.595− −0.008)*	0.90
	Shoal infected	0.170	0.076	(0.022–0.319)*	0.78
	Flow: shoal infected	0.227	0.161	(−0.088–0.542)	0.26
	Standard length	0.007	0.076	(−0.142–0.156)	0.10
Shoal size	(Intercept)	1.167	0.066	(1.138–1.296)	
	Flow	0.102	0.036	(0.032–0.173)*	1.00
	Standard length	0.100	0.036	(0.029–0.170)*	1.00
	Shoal infected	−0.025	0.021	(−0.065–0.016)	0.34
	Individual infected	−0.017	0.023	(−0.061–0.027)	0.14
	Flow: shoal infected	−0.040	0.040	(−0.118–0.037)	0.12
	Flow: standard length	−0.045	0.073	(−0.188–0.098)	0.15
Proportion time shoaling	(Intercept)	1.596	10.652	(−19.282–22.474)	
	Standard length	0.769	0.486	(−0.183–1.721)	0.60
	Individual infected	4.742	161.942	(−312.658–322.142)	0.34
	Flow	1.205	32.555	(−62.601–65.011)	0.28
	Flow: individual infected	16.481	385.141	(−738.381–771.343)	0.19
	Shoal infected	−0.335	0.459	(−1.236–0.566)	0.17

^a^Semicolon (:) indicates interactions

^b^Infections with parasite *Gyrodactylus turnbulli*

^c^Asterisks (*) indicate confidence intervals not bounding zero and therefore considered significant

**Fig. 2:**
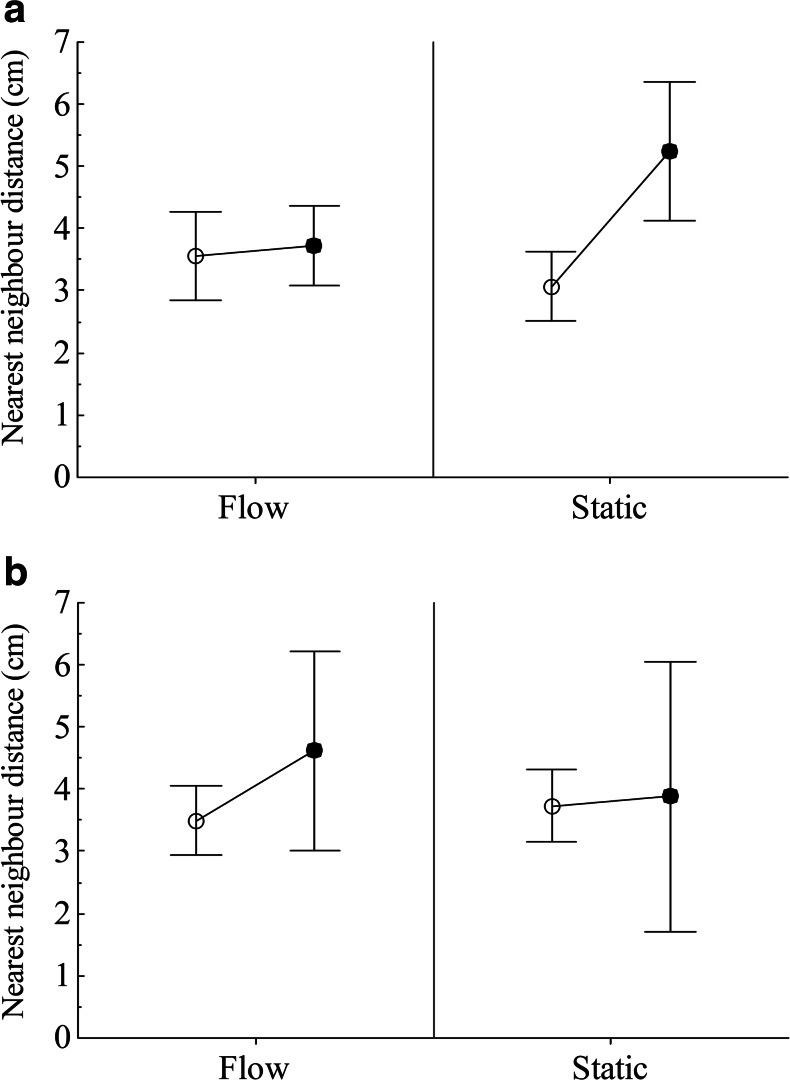
Mean shoal cohesion (nearest neighbour distance) in guppy *Poecilia reticulata* shoals uninfected (*open circles*) and infected (*closed circles*) with *Gyrodactylus turnbulli* in flow and static conditions. *Error bars* show 95 % confidence intervals. Plots in **a** show overall changes in shoal behaviour in response to member being infected and **b** show change in shoaling behaviour for individual infected fish

The interaction between flow condition and infection with *G. turnbulli* was the most important predictor for shoal cohesion (nearest neighbour distance) of the guppy shoals (Table 1). When a member of the group became infected with *G. turnbulli*, there was a reduction in shoal cohesion; however, magnitude of decrease was dependent on flow condition and whether shoaling behaviour was observed at the group or individual level. At the group level, infection with *G. turnbulli* increased mean nearest neighbour distance from mean 3.09 cm (SE 0.32 cm) to 5.17 cm (SE 0.60 cm) in the static condition; this was not observed under flow action with only a minor increase from mean 3.56 cm (SE 0.35 cm) to 3.73 cm (SE 0.30 cm) (Fig. 2a). However, at the individual level, the increase in nearest neighbour distance was less apparent from 3.71 cm (SE 0.33 cm) to 3.89 cm (SE 0.89 cm), whereas under flow action, the increase was much larger from 3.50 cm (SE 0.28 cm) to 4.6 cm (SE 0.62 cm) (Fig. 2b).

During the 5-min observation period, guppies spent a large proportion of their time shoaling (mean 229.63, SE 8.02 s). Larger guppies spent more time shoaling, with standard length having the highest relative importance in the averaged model, although this parameter only appeared in five of the nine top models (relative importance 0.61) and was not considered significant (Table 1). Parasite infection and flow condition had little effect on the time shoaling with 0.35 and 0.29 relative importance, respectively (Table 1).

## Discussion

This is the first study to investigate the interaction between flow condition and parasitism on the shoaling behaviour of fish, and has demonstrated that flow action is an important variable to consider when observing shoaling activity. In the absence of water flow, *P. reticulata* shoals were larger and shoals containing an individual infected with *G. turnbulli* displayed a reduction in shoal cohesion. The size of the fish was also an important predictor for shoaling, with larger fish forming larger shoals in both flow conditions.

The difference in shoal size of *P. reticulata* between the two flow conditions may be explained by energetic allocation. It is more energetically costly to keep up with and maintain large shoals in addition to swimming against a current. When there is no water movement, energy can be allocated to social interaction. In the wild, when there is minimal water movement, predators may also be able to allocate more energy to hunting rather than station holding, and so the guppies would respond to the enhanced predation risk through increased shoaling (Pitcher and Parrish 1993). The effect of flow rate on shoaling has similarly been observed by Garner (1997) and Sneddon et al. (2006) who observed a decrease in shoaling and social hierarchy in minnows and three-spined sticklebacks, respectively, with increasing flow rate. However, the opposite was observed by Allouche and Gaudin (2001) where chub increased aggregation in pools at high flow.

Infection with *G. turnbulli* ectoparasites caused a reduction in guppy shoal cohesion. However the magnitude of this effect differed between the two flow conditions and between the individual and group level. For the infected focal fish, there was an increased nearest neighbour distance between the infected individuals and the rest of the shoal members, which was more apparent under flow action (Fig. 2b). This may be because the infection is preventing the infected fish from keeping up with the rest of the shoal, as suggested by van Oosterhout et al. (2007) where heavily infected individuals were more likely to be washed downstream during flooding events. However, at the group level, the reduction in shoal cohesion between infected and uninfected groups was more apparent in the static condition (Fig. 2a). This may be because in the absence of flow, the infected individual was able to keep up with the shoal and so group respond by increasing the distance to their nearest neighbours to avoid transmission of the parasite. Additionally, in flow, there is a reduced ability of the shoal members to detect the chemical cues of parasitism in flowing water (Archard et al. 2008; James et al. 2008).

Parasitism was not an important predictor of the time spent shoaling and shoal size, suggesting that the antipredator and foraging benefits of shoaling still outweigh the potential costs of shoaling with infected individuals. This result is similar to that found by Richards et al. (2010) who demonstrated a decrease in shoal cohesion with increasing parasite intensity of focal guppies infected with *G. turnbulli* in a static-flow tanks, but no significant difference in the overall time spent shoaling.

The current study found that larger guppies shoaled more by forming larger groups as standard length of the members increased. This is consistent with the findings of Pitcher et al. (1983) who observed larger group sizes of minnow (*P. phoxinus*) and dace (*Leuciscus leuciscus*) with increasing body size. Similarly, Paxton (1996) observed that larger guppies spent more time shoaling. Large fish may be able to benefit from the safety of group living, with the cost of competition for food within the group being lower (at least in the short term) as they are better competitors than smaller individuals (Hoare et al. 2000b). Rodgers et al. (2011) found that only large guppies showed preference for size-matched shoal mates, indicating that shoaling is more important for larger fish as an antipredator response because they are more conspicuous and have a higher calorific value than smaller individuals. The smaller and therefore younger fish may have not yet developed discriminatory shoaling tendencies towards larger groups as demonstrated in Ledesma and McRobert (2008).

It is clear from the current study that flow condition plays an important role in shoaling decisions of fish. Many past laboratory studies have taken place in static tanks where the water remains stationary except for the minimal disturbance to the water by filter systems, as opposed to strong, variable and unidirectional flow action encountered in natural rivers and streams. It is unlikely that in the wild, guppies will experience completely static water, with a small amount of water movement in deeper pools in the stream systems. It would therefore be interesting for further study to compare a range of flow conditions likely experienced by guppies in the wild to determine optimal flow conditions for parasite avoidance and predator detection. Climate-induced changes to hydrology will result in more frequent drought and flooding events (Floury et al. 2013), and therefore, it is important for future study into the behaviour of riverine fish to consider water velocity. Flooding events may also physically wash fish and their parasites (van Oosterhout et al. 2007) into low-flow areas of a river, therefore generating spatial variation in disease prevalence within a river system, which may explain variation in parasite load in wild systems. Flow condition may also play an important role in disease transmission. With an increased shoaling tendency in the static condition, the higher aggregation could lead to more host-host contacts and therefore increased parasite transmission. Although this is yet to be tested in this system, if this were to be the case, there could be important outcomes for disease management in both the aquarium trade and wildlife conservation by habitat manipulation.

## Ethical standards

This work complies with current laws of the UK. All animal work described in the manuscript conforms to the principles of the National Research Council Guide for the Care and Use of Laboratory Animals (2010). It was approved by Cardiff University ethical committee and covered by UK Home Office regulations (PPL 30/2357).
